# Bismuth Shielding in Head Computed Tomography—Still Necessary?

**DOI:** 10.3390/jcm13010025

**Published:** 2023-12-20

**Authors:** Jana Di Rosso, Andreas Krasser, Sebastian Tschauner, Helmuth Guss, Erich Sorantin

**Affiliations:** 1 Division of Paediatric Radiology, Department of Radiology, Medical University of Graz, 8036 Graz, Austriaerich.sorantin@medunigraz.at (E.S.); 2 Competence Centre for Medical Physics and Radiation Protection, University Hospital Graz, 8036 Graz, Austria

**Keywords:** bismuth, radiation, ionizing, radiation protection, lens, crystalline, multidetector computed tomography

## Abstract

**Introduction:** Cranial CT scans are associated with radiation exposure to the eye lens, which is a particularly radiosensitive organ. Children are more vulnerable to radiation than adults. Therefore, it is essential to use the available dose reduction techniques to minimize radiation exposure. According to the European Consensus on patient contact shielding by the IRCP from 2021, shielding is not recommended in most body areas anymore. This study aims to evaluate whether bismuth shielding as well as its combination with other dose-saving technologies could still be useful. **Methods:** Cranial CT scans of a pediatric anthropomorphic phantom were performed on two up-to-date MDCT scanners. Eye lens dose measurements were performed using thermoluminescent dosimeters. Furthermore, the impact of BS and of the additional placement of standoff foam between the patient and BS on image quality was also assessed. **Results:** Bismuth shielding showed a significant lens dose reduction in both CT scanners (GE: 41.50 ± 4.04%, *p* < 0.001; Siemens: 29.75 ± 6.55%, *p* = 0.00). When combined with AEC, the dose was lowered even more (GE: 60.75 ± 3.30%, *p* < 0.001; Siemens: 41.25 ± 8.02%, *p* = 0.00). The highest eye dose reduction was achieved using BS + AEC + OBTCM (GE: 71.25 ± 2.98%, *p* < 0.001; Siemens: 58.75 ± 5.85%, *p* < 0.001). BS caused increased image noise in the orbital region, which could be mitigated by foam placement. Eye shielding had no effect on the image noise in the cranium. **Conclusions:** The use of BS in cranial CT can lead to a significant dose reduction, which can be further enhanced by its combination with other modern dose reduction methods. BS causes increase in image noise in the orbital region but not in the cranium. The additional use of standoff foam reduces image noise in the orbital region.

## 1. Introduction

Medical imaging plays an important role in patient management, including pediatric care. Growth is accomplished through elevated cell turnover, which means that a greater number of cells are in a vulnerable state. Therefore, children exhibit increased radiation sensitivity compared to adults. Due to this increased susceptibility and a long life expectancy post-exposure, children are at a higher risk of radiation-induced damage [1].

In pediatric radiology, the ultrasound and MRI are important methods used in order to avoid the risk of radiation. However, there are circumstances where CT is necessary to provide accurate diagnostic results.

Computed tomography (CT) is a powerful imaging modality widely used in medical diagnostics, especially for examining the brain and other structures within the skull. CT scans are still the main source of medical radiation exposure [2]. CT scans in pediatric radiology account for about 10% of the radiation-based imaging modalities. However, they are responsible for about 40–60% of the radiation exposure in children [1]. The radiation exposure associated with CT scans can cause harmful effects to the patient, especially to the lens of the eye. The lens is particularly sensitive to ionizing radiation, and exposure to even low levels can increase the risk of cataract formation. This risk is particularly concerning for patients who require repeated or frequent CT scans, such as those with head trauma, tumors, or neurological disorders.

To mitigate the risk of radiation-induced cataracts, various strategies have been proposed. Bismuth shielding (BS) has been described as an effective measure in reducing the dose to the eye lenses [3].

Structural shielding (e.g., blanket, rubber mat…) has been used in radiology for the last 70 years [4]. Bismuth shielding was first described by Hopper et al. in 1997 as the use of an in-plane overlying bismuth radioprotective material manufactured into form-fitting garments, which reduces radiation exposure to superficial organs [5]. Since then, shielding has been mainly used in CT to protect eyes, the thyroid gland, and breasts. By placing the shield over radiosensitive organs, the radiation exposition can be reduced by 30–50% [3].

However, in 2021 the International Commission on Radiological Protection (IRCP) released the European Consensus on patient contact shielding, in which it is recommended to discontinue shielding in most body areas including the eye lenses [4]. The main concerns related to shielding are a reduction in image quality and interference with other dose reduction systems, as well as factors related to the operator (inappropriate placement of the shield, infection control) and the patient (patient discomfort, movement during the examination). According to the IRCP, there are other, more effective dose-saving methods that reduce the dose while improving the image consistency, e.g., automatic exposure control systems [4].

This study assesses the eye lens dose and image quality when using BS and its combination with other currently used dose-saving technologies in paediatric cranial CT on two up-to-date multidetector computed tomography (MDCT) scanners. Moreover, it discusses practical considerations for implementing BS in clinical paediatric care and aims to inform its appropriate use in cranial MDCT with modern MDCT scanners.

## 2. Materials and Methods

This phantom study evaluates if there is an additional value of BS used on two modern MDCT scanners equipped with currently used dose reduction technologies, as well as the influence of BS on the image quality in head CT. Since the used CT machines differ considerably in their features and different scanning parameters were used, the radiation exposure differences were compared only for the individual CT machine and not between the scanners.

Ethical committee review and approval were not required because phantom studies were performed.

### 2.1. Phantoms, Bismuth Shield

**Figure 1:**
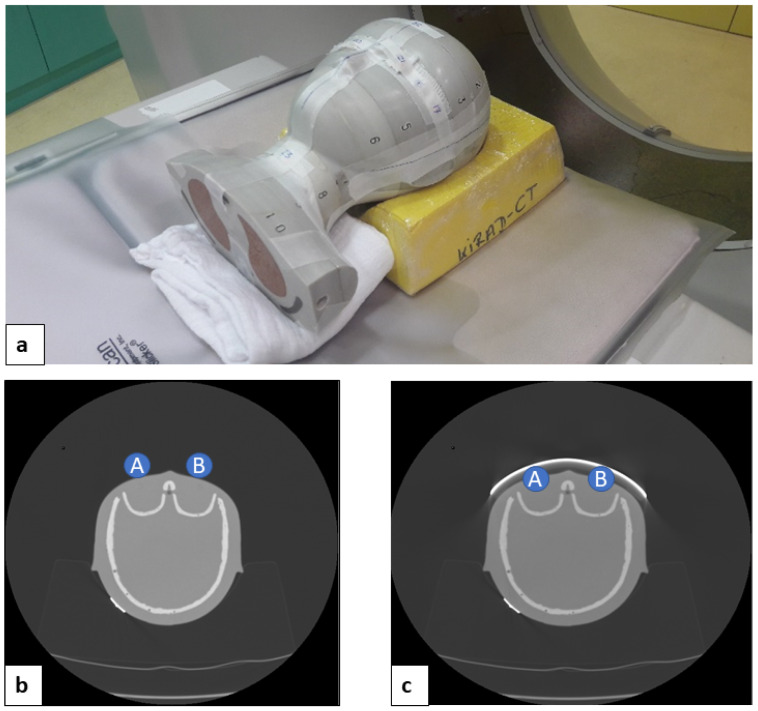
Positioning of the pediatric head phantom within the scanner (**a**) CT axial scans without bismuth shield (**b**) and with bismuth shield (**c**) (measurement points: A, right lens; B, left lens).

An anthropomorphic pediatric phantom (CIRS ATOM^®^ phantom, pediatric 5 years, 110 cm, 19 kg, Computerized Imaging Reference Systems, Inc., Norfolk, VA, USA) was used. After the localizer CT radiograph was acquired, a 1.27 mm thick bismuth shield (14 × 3.5 cm, Somatex, Berlin, Germany) was placed over both phantom lens areas (Figure 1).

### 2.2. MDCT Scanners, Scanning Protocols

The following two up-to-date CT scanners were used for the study: General Electric (GE) Revolution™ (GE, Milwaukee, WI, USA) and Siemens Somatom Definition AS™ (Siemens Healthineers, Erlangen, Germany). Scans on the GE scanner were performed in the helix and volume modes. Furthermore, in the GE scanner, scans in the volume mode with a fixed tube current were acquired. The Siemens scanner provides only the helix mode for the required ranges of this study.

On both scanners, age-adjusted scanning protocols were chosen (Table 1), as used in clinical practice, together with iterative reconstructions.

### 2.3. Dose-Saving Techniques

Automated exposure control (AEC) and organ-based tube current modulation (OBTCM) are modern dose-saving techniques available on the used MDCT scanners. These techniques have vendor-specific characteristics. The GE scanner offers smart mA™ (AEC) and ODM™ (OBTCM). Care Dose 4D™ (AEC) and X-care™ (OBTCM) are provided by the Siemens scanner.

The scans for surface dose and image quality assessment were conducted under different conditions, as shown in Table 2: (a) reference scan without any dose reduction method, (b) AEC, (c) AEC + OBTCM, (d) BS, (e) BS + AEC, (f) BS + AEC + OBTCM.

Automated exposure control (AEC) is a dose reduction technique used to provide automatic adaptation of mA based on the user-specified image quality and X-ray attenuation characteristics of the scanned body region. The goal of the AEC is to apply radiation to the patient more efficiently and keep the image quality constant. Smart mA™ is GE’s technology for automated exposure control. The technology offered by Siemens is called Care Dose 4D™.

Organ-based tube current modulation (OBTCM) is used to reduce the dose delivered to superficial radiosensitive organs in CT by reducing the tube current when the X-ray tube passes over the organs. GE and Siemens have developed different OBTCM techniques called Organ Dose Modulation (ODM™) and X-care™, respectively.

Organ Dose Modulation (ODM™) is offered by GE and it is used to reduce the tube current in a 180° radial arc in body protocols and in a 90° radial arc in head protocols. ODM™ does not increase tube current in other projections, which leads to a reduction in the radiation dose.

X-care™ is Siemens’s technology for organ-based tube current modulation. This dose-saving technique is used to allocate the tube current more to the lateral and posterior tube positions than to the anterior tube positions while keeping the total scanner output the same. It reduces the tube current in a 120° radial arc above the anterior organs and increases the tube current posteriorly in the remaining 240° of the scanning range, so that the radiation dose and image quality remain constant. Superficially located organs have a reduced exposure within the 120° radial arc and increased exposure over the 240°.

### 2.4. Dosimetry

The eye lens dose was estimated using thermoluminescent dosimeters (TLD-100™, Rods 1 × 6 mm, LiF:Mg,Ti, Thermo Scientific™ Waltham, MA, USA) placed over the eye areas of the anthropomorphic pediatric head phantom.

To obtain reasonable dose values, all scans were conducted 15 times, and afterwards measured values were divided by 15 to obtain the dose of an individual scan. The factor 15 was empirically determined in a pre-study. The whole procedure was repeated three times and dose values of dosimeters were averaged.

### 2.5. Image Quality Assessment, ROI Measurements

Image noise represents a marker of image quality. In many studies, increases in image noise and artifacts has been reported as the biggest disadvantage of BS [6].

**Figure 2:**
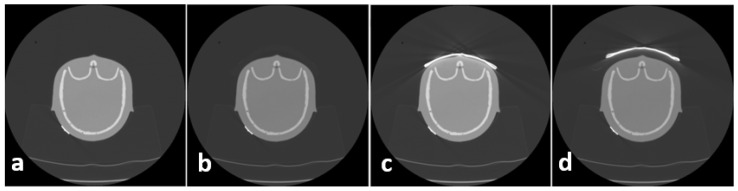
Protocol for quality assessment included 4 scans pro series: phantom (**a**), phantom + foam (**b**), phantom + bismuth shield (**c**), phantom + foam + bismuth shield (**d**).

In order to overcome this disadvantage, the following approach proved to be successful at the authors’ institution: a 2.0 cm standoff foam is placed between the patient and BS to reduce artifacts in superficial areas. To assess the influence of BS as well as the influence of foam on image quality, CT scans were acquired in the following way: phantom alone, phantom + foam, phantom + BS, and phantom + foam + BS, as depicted in Figure 2. These scans were conducted only for the assessment of image quality; therefore, no dosimetric values were acquired.

**Figure 3:**
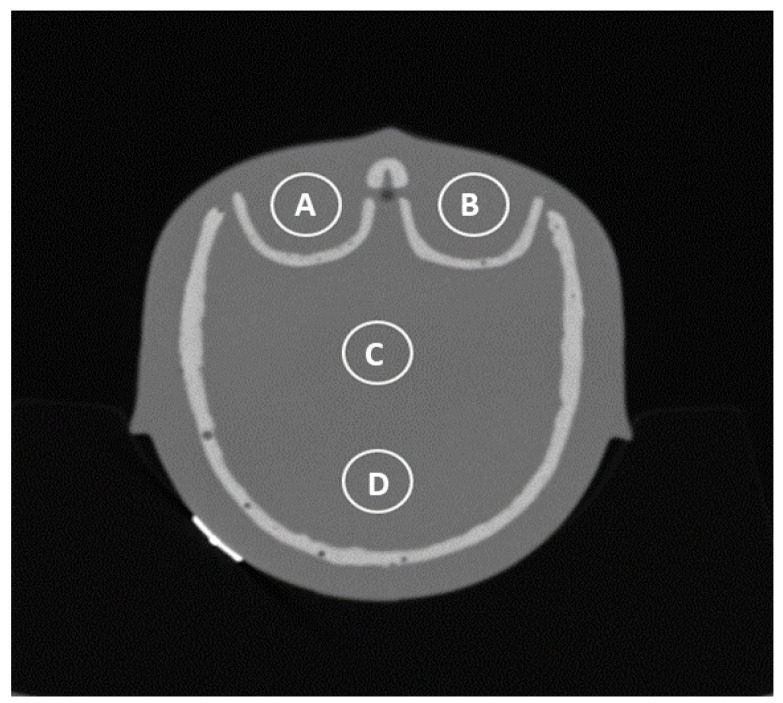
Different points of ROI measurement: orbital right (A), orbital left (B), central in the cranium (C), and occiput (D).

Image noise was estimated using the standard deviation of density values (Hounsfield units, HU) within a circular region of interest (ROI—Figure 3) and served as a quantitative parameter of image quality.

### 2.6. Statistical Analysis

Dose savings were calculated in % based on a scanner reference scan.

*t*-tests, one-way analysis of variance, and post hoc tests were performed. Dose savings under different scanning conditions were compared (reference scan without any dose reduction method, AEC, AEC + OBTCM, BS, BS + AEC, and BS + AEC + OBTCM). Furthermore, the influence of the foam on the image quality was evaluated comparing different imaging conditions (phantom alone, phantom + foam, phantom + BS, phantom + foam + BS).

*p* < 0.05 was considered to indicate a statistically significant difference. The tests were performed in statistical software IBM SPSS Statistics for Windows, Version 21.0 (Armonk, NY, USA: IBM Corp. Released 2012).

## 3. Results

### 3.1. Eye Lens Dose Evaluation

All scan modes showed symmetrical doses between the two eye areas.

AEC led to a statistically significant dose reduction in the GE scanner (21.50 ± 10.28%, *p* = 0.025) but not in the Siemens scanner (−0.75 ± 11.87%, *p* = 0.907).

A dose reduction of over 40% was also achieved without BS, using only the combination of AEC and OBTCM (GE: 50.5. ± 5.07%, *p* < 0.001; Siemens: 43.50 ± 4.36%, *p* < 0.001).

Bismuth shielding alone showed a significant dose reduction (GE: 41.50 ± 4.04%, *p* < 0.001; Siemens: 29.75 ± 6.55%, *p* = 0.003). A further reduction in dose was observed in combinations of BS with other dose-saving methods, such as BS with AEC (GE: 60.75 ± 3.30%, *p* < 0.001; Siemens: 41.25 ± 8.02%, *p* = 0.002). The highest eye dose reduction was achieved using BS + AEC + OBTCM (GE: 71.25 ± 2.98%, *p* < 0.001; Siemens: 58.75 ± 5.85%, *p* < 0.001).

**Figure 4:**
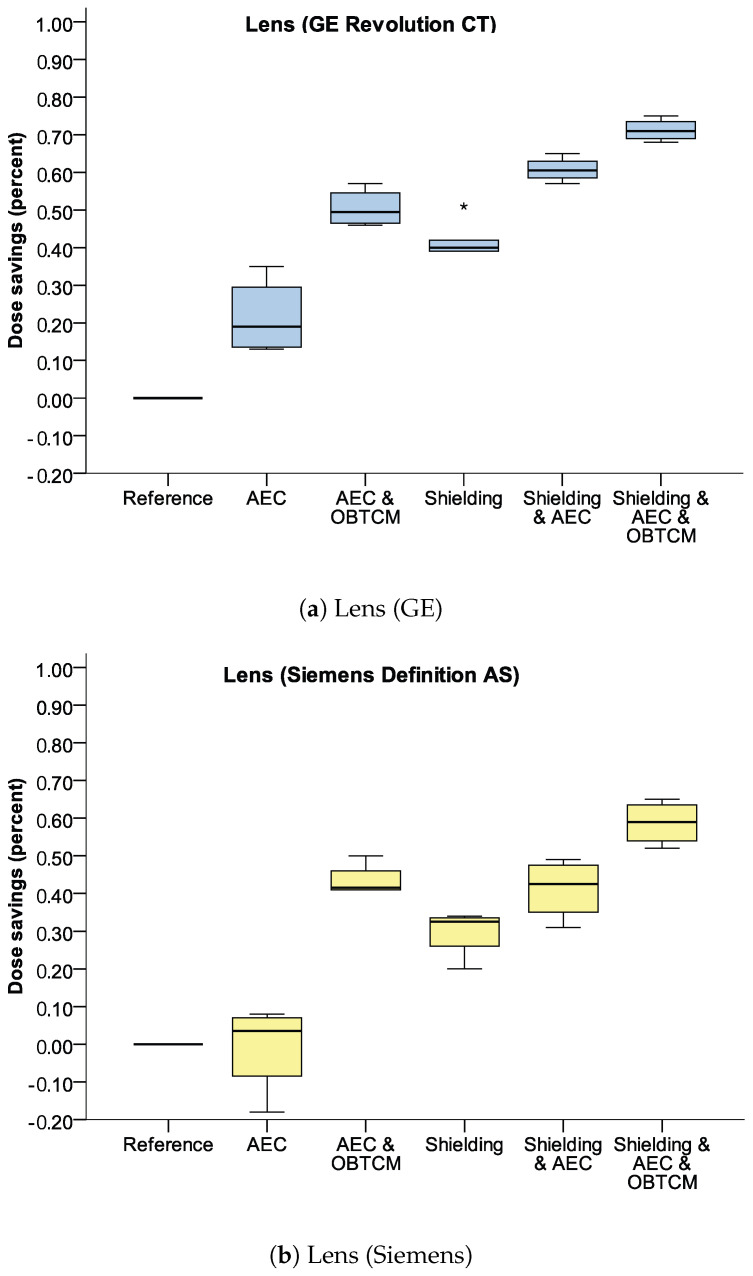
Average relative dose savings of both CT scanners for lens. 1.00 = 100%. Note particularly the difference between AEC and OBTCM without/with shielding. Asterisks (*) represent outliers in the box plot.

The impact of different dose-saving methods and their combination on the dose is shown in Figure 4.

A direct comparison between different dose-saving methods provided by the two different scanners is not possible, since there different scanning parameters were used, as shown previously in Table 2.

### 3.2. Image Quality Assessment

**Figure 5:**
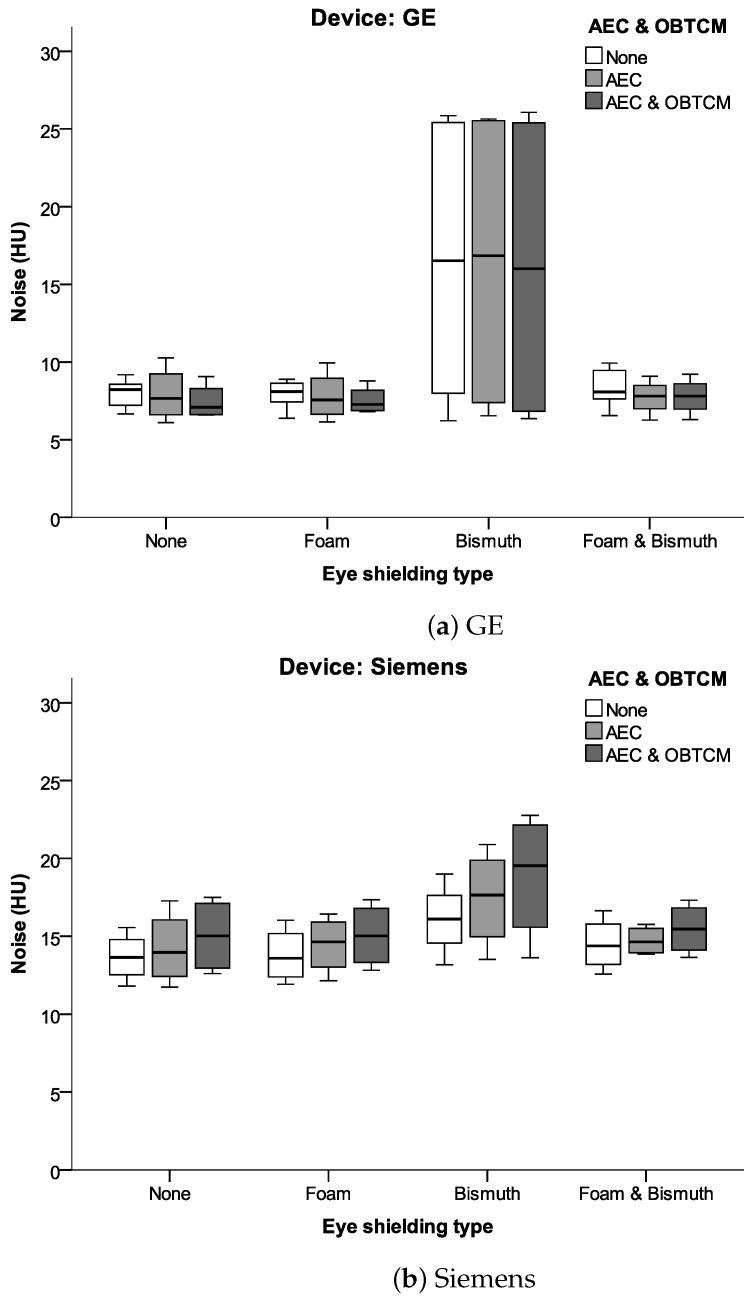
Box plots of mean noise values in the orbital region for different shielding types and different dose reduction methods (AEC and OBTCM). Note the positive effects of placing plastic foam between the bismuth shielding and the eyes.

There was no significant difference in image noise between ROI A and ROI B (orbit right and left). As shown in Figure 5, relative to the reference scan, bismuth shielding caused a significant increase in the image noise in the orbit in both CT scanners. However, the placement of plastic foam between the eye areas and BS lowered the image noise on both CT scanners.

Spacing foam is composed of homogeneous materials with low X-ray attenuation properties; therefore, it should not affect the image quality. Plastic foam placed on the eye areas without the use of bismuth shielding did not influence image quality.

**Figure 6:**
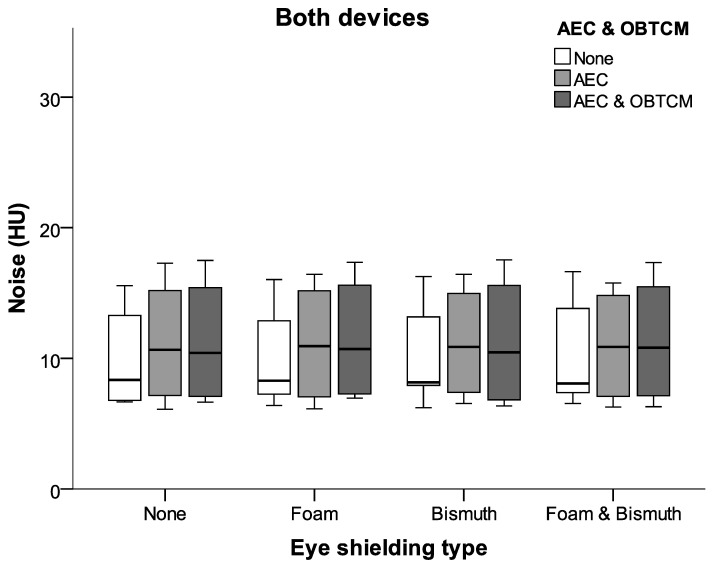
Image noise in the cranium centrally and occipitally for different eye shielding types. Note that shielding apparently had no effect on image noise intracranially.

As shown in Figure 6, eye shielding had no effect on image noise centrally in the cranium and in the occiput, which is crucial for the purpose of brain diagnostics. The additional use of foam in these regions did not lead to a significant decrease in the image noise.

Different scan modes (volume, helix) and dose-saving techniques (AEC, AEC + OBTCM) did not influence the image quality related to shielding.

## 4. Discussion

### 4.1. Results of This Study vs. Current Literature

Bismuth shielding is a practical and effective method for reducing radiation exposure to the eye lens during cranial CT imaging. In the presented study, BS showed a significant lens dose reduction in both CT scanners, which could be enhanced by its combination with other dose reduction techniques. The highest eye dose reduction of up to 70% was achieved using BS + AEC + OBTCM.

Relative to the reference scan, BS led to a significant increase in the image noise in the orbital region. However, the combination of BS with foam lowered the image noise in the orbit significantly. Shielding, as well as its combination with foam, had no effect on image noise intracranially. These results are compatible with the results published in the currently available literature.

A meta-analysis of the effects of BS for the protection of superficial radiosensitive organs from 2019 included pediatric and adult anthropomorphic phantoms, as well as patients undergoing CT. The fixed-effects pooled estimate of eye lens dose reduction was 34%. Shielding was recommended in 88.89% of the studies [6].

There is a study from 2012 that combined BS with OBTCM (XCare™, Siemens) for dose reduction to the eye in head CT. The dose to the eye was reduced by 26.4% with bismuth shielding. A combination of OBTCM with one bismuth shield reduced the dose by 47.0%, but the CT number accuracy and image quality (noise and artifacts) were affected. The image noise in the brain region was slightly increased for all dose reduction methods [3].

In a study from 2020, the effect of barium sulfate and bismuth-antimony shields on the dose reduction to the eye lens and image quality in cranial CT on a MDCT scanner (Somatom Definition Flash; Siemens Healthcare) was investigated. It showed a significant lens dose reduction (by 28.60–31.92% and 43.87–47.00%, respectively) while causing substantial image artifacts. Shielding combined with AEC did not exhibit a significant difference compared to the dose measured under the fixed tube current (FTC). In comparison to a fixed tube current, employing AEC yielded improved signal-to-noise and contrast-to-noise ratios within the intracranial structures when using the bismuth-antimony and barium sulfate shields, respectively [7].

In the available literature, the use of eye shielding has been associated with a moderate increase in image noise of about 1–2 HU. The additional use of foam has led to a decrease in the image noise near the orbit but not in the brain region [3].

To the best of the authors’ knowledge, no current study has been performed evaluating the use of BS and its combination with other currently available dose reduction techniques and its effects on the dose reduction and image quality on up-to-date MDCT scanners.

### 4.2. IRCP Statement

According to the European Consensus on patient contact shielding released in 2021, shielding is not recommended anymore to protect eye lens in cranial CT [4]. However, it might still be used to protect the breast and thyroid if the potential benefit of its use could outweigh the risks, e.g., in numerous examinations and interventional procedures, where the cumulative dose is high. Anxious and radiosensitive patients could also benefit from the use of shielding [6].

### 4.3. Limitations of BS and Potential Solutions

When it comes to eye lens shielding with bismuth in cranial CT, there are several problematic points and limitations that need to be considered, especially in children:

- Dose reduction: While bismuth shields are effective in reducing the radiation dose to the eye lens, they do not eliminate it entirely. Moreover, the shielding effect is highly dependent on the design and placement of the shield. In children, who are more susceptible to radiation-induced damage than adults, it is important to balance the benefits of dose reduction with the potential risks associated with additional imaging.
- Image artifacts: BS effects the image quality due to increased image noise and streak artifacts. Artifacts may interfere with the interpretation of CT images and can be especially problematic in the context of pediatric CT, where image quality is critical for accurate diagnosis and treatment planning. Foams have been used to increase the distance between the bismuth shield and the superficial structures, reducing the image noise and artifacts under shielding [6], which also proved to be useful in the presented study.
- Interference with the automatic exposure control systems: This can lead to increased radiation. Therefore, the shield should be placed after acquiring the scout view, as conducted in the presented study [6].
- Practical considerations: Bismuth shields can be difficult to apply in pediatric patients, who may be more prone to movement and require specialized techniques to maintain a stable head position during imaging. In addition, the use of bismuth shields may require additional time and effort on the part of the technologist (placement of the shield, infection control), which can increase the overall imaging time.
- Patient comfort: Children may be uncomfortable with the use of bismuth shields, which can be heavy and difficult to position. Moreover, the use of shields may increase anxiety or fear, leading to more difficulties in imaging or even sedation.

### 4.4. Alternative Techniques

There are alternative techniques that can be used to reduce radiation the dose to the eye lens in cranial CT, such as automatic exposure control systems, low-dose protocols, globally lowering the tube current (GLTC), reducing the tube voltage (RTV), or gantry tilting [8].

Gantry tilting is one of the most effective ways to protect the eye lens in head CT but it is not always possible in pediatric radiology, due, e.g., to patient positioning limitations or contraindications of head tilting.

However, the effectiveness of these techniques may vary depending on the patient and the clinical indication, and bismuth shielding may still be necessary in some cases.

### 4.5. Optimization of Bismuth Shielding Utilization

Overall, the use of bismuth shields for eye lens protection in pediatric CT requires careful consideration of the potential risks and benefits, as well as the practical limitations and patient comfort. The decision to implement shielding at individual institutions should involve a collaborative effort by a multidisciplinary team, including medical physics experts. Furthermore, it should be documented in the examination protocols.

Comprehensive training for staff should encompass proper shielding usage, effective communication with patients, shield selection, and precise positioning, as well as procedures for the cleaning, disinfection, and storage of BS.

Clinical decisions should be made based on the individual patient’s clinical indication, age, size, and imaging characteristics. The use of BS should be optimized to balance the benefits of radiation dose reduction with the potential risks associated with image quality deterioration and additional imaging.

### 4.6. Limitations of This Study

The phantom used in this study represented only the head of a 5-year-old child. At this age, skull calcification is already complete; therefore, the results of this study should also be applicable to older children. X-ray beam attenuation in a less calcified skull of younger children is more similar to that of a toddler torso, which has been evaluated in previous studies and showed similar results [9].

The presented phantom study had a small sample size. Extensive research with patients is necessary, especially regarding image quality under BS and its influence on specific diagnostic tasks.

Dose assessment was limited to the eye lens area because it is a particularly radiosensitive organ and its repeated exposure to radiation is associated with cataract formation.

## 5. Conclusions

The results of this study suggest that bismuth shielding can be beneficial in clinical practice using up-to-date MDCT scanners. Bismuth shielding has proven useful, especially in combination with other dose reduction methods, such as AEC and OBTCM. BS leads to an increase in the image noise in the orbital region, which can be significantly decreased by the placement of plastic foam between the shield and the eyes. Shielding had no effect on image noise intracranially, which is crucial for brain diagnostics.

## Figures and Tables

**Table 1:** Scanning protocols.

CT Scanner	GE Revolution™				Siemens Somatom Definition AS™		
**Scan mode**	Volume	Helix	Helix	Helix	Helix	Helix	Helix
**Tube voltage (kV)**	100	100	100	100	100	100	100
**kV modulation**	-	-	-	-	-	-	-
**Average tube current (mA)**	300	230	223	184	253	192	189
**AEC (z-direction)**	-	-	yes	yes	-	yes	yes
**OBTCM (x,y-direction)**	-	-	-	yes	-	-	yes
**Slice thickness (mm), collimation (rows)**	0.625	0.625 × 40	0.625 × 80	0.625 × 80	0.6 × 40	0.6 × 40	0.6 × 128
**Scan length (mm)**	157.5	160	160	160	160	160	160
**Pitch**	0	0.516	0.507	0.507	0.55	0.55	0.55
**Rotation time (s)**	0.5	0.5	0.5	0.5	0.5	1	1
**Adaptive dose shield**	-	-	-	-	yes	yes	yes
**FOV**	250 mm	250 mm	250 mm	250 mm	250 mm	250 mm	250 mm
**Focus**	0.6 mm	0.6 mm	0.6 mm	0.6 mm	0.7 mm	0.7 mm	0.7 mm
**CTDIvol (mGy)**	12.04	19.15	16.34	13.64	24.08	19.96	16.81
**DLPvol (mGy × cm)**	192.6	384.6	261.5	223.6	401.0	332.6	291.2
**Filtering**	4.2 mm Al				6.8 mm Al		

**Table 2:** Different scanning conditions used for eye lens dose and image quality evaluation.

CT Machine	Scan Mode	Dose Modulation
**GE Revolution™**	Volume	−
	Helix	−
	Helix	+ smart mA
	Helix	+ smart mA + ODM
**Siemens Somatom Definition AS™**	Helix	−
	Helix	+ Care Dose 4D
	Helix	+ Care Dose 4D + X-care

## Data Availability

Data can be obtained from the corresponding author (ST) on request.
